# Incidence and Predictors of Venous Thromboembolism Following Major Urologic Cancer Surgery: Toward Risk-Stratified, Personalized Prophylaxis Strategies

**DOI:** 10.3390/jpm16020082

**Published:** 2026-02-01

**Authors:** Sri Saran Manivasagam, Alireza Aminsharifi, Jay D. Raman

**Affiliations:** Department of Urology, Penn State Health Milton S. Hershey Medical Center, Hershey, PA 17033, USA; smanivasagam@pennstatehealth.psu.edu (S.S.M.); aaminsharifi@pennstatehealth.psu.edu (A.A.)

**Keywords:** deep vein thrombosis, pulmonary embolism, thromboprophylaxis, urology cancer surgery

## Abstract

**Background/Objectives**: Venous thromboembolism (VTE), including deep vein thrombosis (DVT) and pulmonary embolism (PE), remains a significant postoperative complication following major urologic cancer surgeries. Despite widespread use of thromboprophylaxis, the real-world effectiveness of these strategies remains uncertain. **Methods**: We conducted a retrospective cohort study using the American College of Surgeons National Surgical Quality Improvement Program (ACS NSQIP) database, including procedure-targeted data for radical cystectomy, radical prostatectomy, and radical nephrectomy from 2019 to 2022. Patients aged 18–90 years with complete data were included. Descriptive statistics and multivariate logistic regression analyses were performed to identify predictors of DVT and evaluate the impact of thromboprophylaxis strategies. **Results**: A total of 65,105 patients were analyzed: 28,805 prostatectomies, 28,414 cystectomies, and 7886 nephrectomies. The 30-day incidence of DVT and PE was 1.1% and 0.8%, respectively. Multivariate analysis identified prolonged hospital stay (>4 days), operative time (>180 min), and age > 75 years as independent predictors of DVT. Subgroup analyses confirmed these findings for cystectomy and prostatectomy but not for nephrectomy. Thromboprophylaxis was administered in 97.8% of patients; however, its use was not significantly associated with reduced DVT incidence, except for pharmacologic prophylaxis in cystectomy patients (OR 0.04, *p* = 0.03). **Conclusions**: Despite high adherence to thromboprophylaxis protocols, DVT remains a clinically relevant complication after urologic cancer surgery. Our findings highlight the importance of procedural factors in DVT risk and question the universal effectiveness of current prophylaxis strategies. These findings underscore the need for personalized, risk-stratified thromboprophylaxis protocols tailored to patient and procedural factors.

## 1. Introduction

Venous thromboembolism (VTE), encompassing deep vein thrombosis (DVT) and pulmonary embolism (PE), remains a significant postoperative complication in patients undergoing urologic cancer surgery. The 30-day incidence of DVT following urologic procedures is approximately 0.76%; the rate is higher following oncologic surgeries (about 1%), with radical cystectomy presenting the highest risk at 3.96% [1,2,3].

Several factors contribute to the elevated risk of VTE in this patient population, including advanced age, malignancy, and surgical intervention. Additional comorbidities—such as obesity, congestive heart failure, diabetes mellitus (DM), chronic obstructive pulmonary disease (COPD), and recent chemotherapy—further exacerbate this risk [4,5,6,7]. Progression from DVT to PE occurs in approximately 2.5% of cases and is associated with a marked increase in mortality, with in-hospital and 30-day mortality rates reported at 6% and 9.3%, respectively [7,8].

Even among patients who receive successful treatment for DVT, 33% to 50% may develop post-thrombotic syndrome, a condition associated with substantial long-term morbidity. The treatment of established VTE carries inherent risks, including thrombocytopenia and major bleeding events such as intracerebral hemorrhage. For instance, warfarin therapy is associated with a 1.8% incidence of intracranial bleeding. Given these risks, effective thromboprophylaxis is essential in mitigating VTE-related morbidity and mortality [8].

Existing guidelines on perioperative thromboprophylaxis vary considerably across regions, with differences in recommended duration, risk stratification, and procedural specificity. [9,10,11]. Unlike other societies, the American Urological Association (AUA) does not currently offer formal, society-level guidelines for perioperative thromboprophylaxis across all major urologic cancer surgeries. However, individual studies and institutions have been published. This variability in guideline availability and content has led to significant heterogeneity in clinical practice worldwide, including differences in prophylactic strategies, pharmacologic agents, and adherence rates [12,13,14,15]. The advent of direct oral anticoagulants (DOACs), such as apixaban, has further diversified pharmacologic approaches to thromboprophylaxis [16,17,18]. Personalized medicine approaches, which tailor prophylaxis based on individual risk profiles, may optimize outcomes compared to uniform protocols.

The present study aims to update the incidence and identify predictors of DVT following radical cystectomy, radical prostatectomy, and radical nephrectomy. Additionally, it seeks to evaluate the impact of comorbid conditions, operative factors, and thromboprophylaxis strategies using data from the American College of Surgeons National Surgical Quality Improvement Program (ACS NSQIP) database.

## 2. Materials and Methods

### 2.1. Patients

We included patients aged 18–90 years who underwent radical cystectomy, radical prostatectomy, or radical nephrectomy between 2019 and 2022 in the ACS NSQIP procedure-targeted database. Eligibility was based on CPT codes for these procedures. Cases of partial nephrectomy and records with incomplete data were excluded.

### 2.2. Ethical Considerations

This study was exempt from institutional review board approval because it used de-identified ACS NSQIP data. Patient consent was not required.

### 2.3. Study Outcomes

- **Primary Outcome:** 30-day incidence of deep vein thrombosis (DVT) following major urologic cancer surgery. Deep venous thrombosis was defined as a new diagnosis of a blood clot (thrombus) within the deep venous system, which may be accompanied by inflammation and require treatment. Must be noted within 30 days after the principal operative procedure AND one of the following (A or B): A. New diagnosis of a deep venous thrombosis, confirmed by a duplex, venogram, computed tomography (CT) scan, or any other definitive imaging modality (including direct pathology examination such as autopsy) AND the patient must be treated with anticoagulation therapy and/or placement of a vena cava filter or clipping of the vena cava, or the record indicates that treatment was warranted without additional appropriate treatment option available. B. As per (A) above, but the patient or decisionmaker has refused treatment. There must be documentation in the medical record of the refusal of treatment.
- **Secondary Outcomes:** Incidence of pulmonary embolism (PE), identification of independent predictors of DVT, and evaluation of the association between thromboprophylaxis strategies and DVT risk. Pulmonary embolism was defined as: Lodging of a blood clot in the pulmonary artery with subsequent obstruction of blood supply to the lung parenchyma. Blood clots usually originate from the deep leg veins or the pelvic venous system. The identification of a new blood clot in a pulmonary artery causes obstruction (complete or partial) of the blood supply to the lungs. A pulmonary embolism must be noted within 30 days after the principal operative procedure AND the following criteria, A AND B below: A. New diagnosis of a new blood clot in a pulmonary artery AND B. The patient has a ventilation-perfusion scan interpreted as high probability of pulmonary embolism, a positive CT exam, transesophageal echocardiogram, pulmonary arteriogram, CT angiogram, or any other definitive imaging modality (including direct pathology examination, such as autopsy).

### 2.4. Study Design and Definitions

This was a nationwide retrospective cohort study using ACS NSQIP, a validated, risk-adjusted program that collects over 150 variables, including preoperative risk factors, intraoperative details, and 30-day postoperative outcomes. The data used in this study were obtained from the ACS NSQIP and participating hospitals. However, the American College of Surgeons and the participating hospitals have neither verified nor are responsible for the statistical analyses or conclusions presented in the manuscript.

### 2.5. Statistical Analysis

Descriptive statistics summarized patient characteristics. Continuous variables were reported as mean ± SD; categorical variables as frequencies and percentages. Chi-square tests compared categorical variables. Multivariate logistic regression analyses were performed to identify predictors of deep venous thrombosis. The following variables were included in the models: age > 75 years, body mass index (BMI) > 35 kg/m^2^, American Society of Anesthesiologists (ASA) Classification System 3–5, smoking status, diabetes mellitus, hypertension, COPD, congestive heart failure, dependent functional status, operative time > 180 min, open surgical approach, length of hospital stay > 4 days, and type of thromboprophylaxis (mechanical, pharmacological, combined, or no prophylaxis). Cutoffs for age (>75 years), operative time (>180 min), BMI (>35 kg/m^2^), and length of stay (>4 days) were selected based on established DVT risk factors reported in prior studies, NSQIP standards, and the distribution of these variables in our dataset. All models were tested for goodness-of-fit using the Hosmer–Lemeshow test and for multicollinearity using variance inflation factors. Analyses were performed using IBM SPSS Statistics v30, with *p* < 0.05 considered significant. No generative AI was used in the development of this manuscript.

## 3. Results

A total of 65,105 patients who underwent radical cystectomy, radical prostatectomy, or radical nephrectomy between 2019 and 2022 were included in the analysis. The cohort comprised 28,805 radical prostatectomy cases (44.2%), 28,414 radical cystectomy cases (43.6%), and 7886 radical nephrectomy cases (12.1%).

The overall 30-day incidence of deep vein thrombosis (DVT) was 1.1%, while PE occurred in 0.8% of patients. Among the three procedures, radical cystectomy was associated with the highest incidence of both DVT (1.92%) and PE (1.24%). In comparison, the incidence of DVT was 0.48% following radical prostatectomy and 0.44% following nephrectomy. Similarly, PE occurred in 0.47% of radical prostatectomy cases and 0.42% of nephrectomy cases (Table 1).

The median time to diagnosis was 10 days for DVT and 14 days for PE. Thromboprophylaxis was administered in 97.8% of patients: 75.9% received both mechanical and pharmacologic prophylaxis, 17.9% received mechanical prophylaxis only, 4.0% received pharmacologic prophylaxis only, and 2.2% received no prophylaxis.

### 3.1. Baseline Characteristics

Patients who developed DVT demonstrated distinct clinical characteristics compared to those who did not. They were significantly older (mean age: 70.0 vs. 66.3 years; *p* < 0.001) and had a higher prevalence of comorbid conditions, including insulin-dependent diabetes mellitus (8.2% vs. 5.5%; *p* < 0.001), COPD (5.7% vs. 3.8%; *p* < 0.001), congestive heart failure (2.9% vs. 1.2%; *p* < 0.001), hypertension (53.5% vs. 47.5%; *p* < 0.001), and chronic kidney disease requiring dialysis (2.2% vs. 1.1%; *p* < 0.001).

A higher proportion of patients who developed DVT were classified as ASA grade 3–5 (74% vs. 53%; *p* < 0.001), a higher percentage were functionally independent (97% vs. 93.9%; *p* < 0.001), and underwent open surgical procedures (58.6% vs. 32.1%; *p* < 0.001). These patients also experienced significantly longer operative times (mean: 332.9 min vs. 259.4 min; *p* < 0.001) and extended hospital stays (mean: 9.7 days vs. 4.4 days; *p* < 0.001).

Interestingly, a higher proportion of patients who developed DVT had received both mechanical and pharmacologic prophylaxis compared to those who did not (86.3% vs. 75.7%; *p* = 0.011), suggesting potential confounding by indication or differences in baseline risk profiles (Table 2).

### 3.2. Regression Analysis

Multivariate logistic regression was performed to identify independent predictors of DVT, adjusting for age > 75 years, body mass index (BMI) > 35 kg/m^2^, ASA physical status classification (grades 3–5), current smoking status, dependent functional status, operative time > 180 min, surgical approach, length of hospital stay > 4 days, and thromboprophylaxis strategies (Table 3).

In the overall cohort, three factors were independently associated with an increased risk of DVT:

- Length of hospital stay > 4 days: OR 2.63, 95% CI: 1.78–3.96, *p* < 0.001
- Operative time > 180 min: OR 2.20, 95% CI: 1.45–3.35, *p* < 0.001
- Age > 75 years: OR 1.70, 95% CI: 1.07–2.71, *p* = 0.025

Subgroup analyses by surgical procedure revealed consistent findings:

- Radical prostatectomy cohort:
  ○ Length of stay > 4 days: OR 2.96, 95% CI: 1.23–7.10, *p* = 0.015
  ○ Operative time > 180 min: OR 2.03, 95% CI: 1.18–3.48, *p* = 0.01
  ○ Diabetes Mellitus: OR 1.79, 95% CI: 1.06–3.04, *p* = 0.029
- Radical cystectomy cohort:
  ○ Length of stay > 4 days: OR 5.37, 95% CI: 1.90–15.20, *p* = 0.004
  ○ Operative time > 180 min: OR 3.38, 95% CI: 1.01–11.21, *p* = 0.047
  ○ Age > 75 years: OR 1.53, 95% CI: 1.09–2.15, *p* <0.001

In contrast, none of the above predictors was statistically significant in the radical nephrectomy cohort, potentially due to lower event rates.

Interestingly, across the overall cohort, thromboprophylaxis strategies (combined, mechanical-only, or pharmacologic-only) did not demonstrate a statistically significant reduction in DVT risk. However, in the cystectomy subgroup, pharmacologic prophylaxis alone was associated with a significantly lower risk of DVT (OR 0.04, 95% CI: 0.003–0.83, *p* = 0.037) (Table 3).

## 4. Discussion

In this large database study, we estimated the 30-day incidence of DVT and PE and their associated risk factors following major urologic cancer surgeries. The incidence rates of DVT and PE were 1.1% and 0.8%, respectively. Despite high adherence to thromboprophylaxis (97.8%), DVT remains a persistent and clinically relevant complication in this surgical population. Notably, thromboprophylaxis strategies did not demonstrate a statistically significant reduction in overall DVT risk across the entire cohort, raising important questions about the clinical effectiveness of current prophylaxis protocols. Multivariate regression analysis revealed that DVT risk is significantly influenced by patient age, operative factors, and specific comorbidities.

Our estimated DVT incidence aligns with previously published data [1,2,3,19]. Most prior studies have focused on identifying risk factors for DVT. For instance, a retrospective review by Elsayed et al. (2020) found that higher body mass index (BMI) and chronic obstructive pulmonary disease (COPD) were associated with increased DVT risk following radical cystectomy [20]. Similarly, Najdi et al., in their review of the ACS NSQIP database (2011–2020), reported that advanced age, BMI > 40 kg/m^2^, and congestive heart failure were significantly associated with DVT risk [3]. Our findings are consistent with these results, identifying advanced age as a significant predictor of DVT.

A particularly noteworthy observation in our study is the near-universal use of thromboprophylaxis (97.8%). Mechanical methods included sequential compression devices, compression stockings, and early ambulation within 24 h. Pharmacologic prophylaxis typically involves low-molecular-weight heparins (LMWH), fondaparinux, or direct oral anticoagulants (DOACs). Despite this, DVT events continue to occur, raising important concerns about the consistency, duration, and perhaps the adequacy of current thromboprophylaxis protocols [21]. The observation that a higher proportion of patients who developed DVT had received both mechanical and pharmacologic prophylaxis compared to those who did not is likely explained by confounding by indication. Patients perceived as the highest risk due to advanced age, comorbidities, or complex surgery were more likely to receive combined prophylaxis. These same factors independently increase DVT risk, potentially overshadowing the protective effect of combined prophylaxis in multivariate models. Additionally, mechanical methods may have been inconsistently applied or discontinued early postoperatively, reducing their additive benefit. This paradox underscores the limitations of observational data and highlights the need for prospective studies to disentangle true prophylactic efficacy from baseline risk.

In the radical cystectomy subgroup, pharmacologic prophylaxis alone was associated with a markedly lower risk of DVT (OR 0.04, 95% CI: 0.002–0.83, *p* = 0.03). While this suggests a strong protective effect, interpretation is limited by the small size of this group and the resulting wide confidence interval. Residual confounding is also possible, as patients receiving pharmacologic-only prophylaxis may have differed in baseline risk compared to those receiving combined prophylaxis. These findings highlight the need for prospective studies to clarify whether pharmacologic-only strategies truly outperform combined approaches in high-risk surgeries.

The optimal duration of thromboprophylaxis remains a subject of ongoing debate. Naik et al., in a meta-analysis of 18 studies, concluded that extended thromboprophylaxis (ETP) for 28 days significantly reduced DVT incidence, though it did not affect PE rates or overall mortality [22]. Others have developed a risk-stratified, evidence-based decision aid to guide ETP use, emphasizing a patient-centered approach, highlighting the importance of ETP in reducing DVT following radical cystectomy [23,24].

Our findings underscore the importance of both patient- and procedure-specific factors in DVT development, even in the presence of prophylaxis. This prompts further inquiry: Are high-risk patients receiving adequate prophylaxis in terms of duration and intensity? Conversely, are low-risk patients being exposed to unnecessary bleeding risks due to overuse of anticoagulation? These considerations highlight the need for patient-centered thromboprophylaxis guidelines.

Several risk assessment models (RAMs) have been proposed to guide prophylaxis decisions. Douroumis et al. (2025) compared the European Association of Urology (EAU), American Urological Association (AUA), and Caprini RAMs, finding that the Caprini score often led to excessive prophylaxis recommendations, while the EAU model was more user-friendly but required cautious application due to bleeding risks [25]. Klaassen et al. proposed a risk-based approach for extended prophylaxis after prostatectomy, incorporating four factors: age > 75 years, BMI > 35 kg/m^2^, a first-degree relative with DVT, and personal history of DVT. They emphasized the importance of balancing prophylactic benefits against bleeding risks [26].

Our study contributes to the literature by evaluating the real-world effectiveness of thromboprophylaxis in a large, diverse cohort of 65,105 patients undergoing major urologic cancer surgeries. The multivariate regression models, combined with the granularity of patient-level data from the ACS NSQIP database, improve the internal validity of our findings. Additionally, the inclusion of a wide range of urologic procedures and the stratified analysis enhances the generalizability of our results across different surgical contexts. Our study is the first to analyze the clear impact of thromboprophylaxis measures in the prevention of DVT. Our results challenge the one-size-fits-all approach and highlight the importance of personalized prophylaxis strategies informed by patient-specific and procedural risk factors.

However, several limitations must be acknowledged. First, the retrospective nature of the study introduces the potential for selection bias and residual confounding, despite multivariate adjustment. Second, the ACS NSQIP database, while comprehensive, lacks detailed information on the specific pharmacologic agents used, their dosages, timing, and duration of administration—all of which may influence DVT outcomes. Third, we were unable to assess adherence to prophylaxis protocols beyond the perioperative period, nor could we evaluate patient-level factors such as mobility status or genetic predisposition to thrombosis. Additionally, the database does not capture post-discharge DVT events beyond 30 days, which may underestimate the true incidence of late thromboembolic complications, particularly in high-risk populations.

Finally, while our findings suggest differential impacts of prophylaxis strategies across surgical subtypes, causality cannot be established given the observational design. Prospective, randomized studies are needed to validate these associations and to refine risk-stratified, patient-centered prophylaxis protocols in urologic oncology.

## 5. Conclusions

This retrospective cohort study provides updated estimates of DVT and PE incidence following major urologic cancer surgeries and underscores the persistent burden of DVT despite widespread prophylaxis. We demonstrate that advanced age, prolonged operative time, and extended hospitalization are independent predictors of DVT. Moreover, the significant reduction in DVT risk associated with pharmacologic prophylaxis in the cystectomy subgroup highlights the potential for procedure-specific tailoring of prophylactic strategies.

These results challenge the current uniform approach to perioperative thromboprophylaxis and support the development of risk-stratified, evidence-based protocols customized to the surgical context and patient profile. Future research should focus on developing and validating personalized, risk-stratified prophylaxis guidelines to improve patient safety and outcomes.

## Figures and Tables

**Table 1:** Incidence of DVT and PE across various procedures.

Procedure	DVT N, %	PE N, %
Radical cystectomy	545, 1.92%	354, 1.24%
Radical nephrectomy	35, 0.44%	33, 0.42%
Radical prostatectomy	137, 0.48%	134, 0.47%
All cases	717, 1.1%	521, 0.8%

DVT = Deep Vein Thrombosis; PE = Pulmonary Embolism.

**Table 2:** Distribution of clinical and operative variables and prophylaxis strategies across the cohort stratified by the development of DVT.

	No DVT (*n* = 64,388)	DVT (*n* = 717)	*p*-Value
Age in years Mean, SD	66.33, 9.283	70.05, 9.349	<0.001
BMI in kg/m^2^ Mean, SD	30.18, 6.19	30.54, 6.54	0.223
% DM, requiring Insulin	3541, 5.5%	59, 8.2%	<0.001
% Current smoker	9400, 14.6%	104, 14.5%	0.933
%Functional status, independent	60,460, 93.9%	696, 97%	<0.001
% History of COPD	2446, 3.8%	41, 5.7%	<0.001
% History of CHF	772, 1.2%	21, 2.9%	<0.001
% History of Hypertension	30,584, 47.5%	384, 53.5%	<0.001
% CKD, On Dialysis	708, 1.1%	16, 2.23%	<0.001
ASA 3/4/5	34,125, 53%	530,74%	<0.001
% Open Surgery	20,668, 32.1%	420, 58.57%	<0.001
Operation time (Min) Mean, SD	259.42, 111.9	332.90, 133.9	<0.001
Length of hospital stay (days) Mean, SD	4.383, 3.28	9.73, 7.1	<0.001
Prophylaxis strategy:			
Mechanical and pharmacologic prophylaxis %	48,741, 75.7%	619, 86.3%	
Mechanical prophylaxis, %	11,589, 18%	78, 10.8%	0.011
Pharmacologic prophylaxis, %	2641, 4.1%	8, 1.1%	
No prophylaxis	1417, 2.2%	12, 1.6%	

DVT = Deep vein thrombosis, BMI = Body Mass Index; DM = Diabetes Mellitus; COPD = Chronic Obstructive Pulmonary Disease; CHF = Congestive Heart Failure; CKD = Chronic Kidney Disease; ASA = American Society of Anesthesiologists Classification System.

**Table 3:** Multivariate logistic regression for prediction of DVT following urologic cancer surgery.

	All Cases		Radical Prostatectomy		Radical Cystectomy		Radical Nephrectomy	
	Odds Ratio (95% CI)	*p*-Value	Odds Ratio (95% CI)	*p*-Value	Odds Ratio (95% CI)	*p*-Value	Odds Ratio (95% CI)	*P*-Value
Age > 75 years	1.705 (1.071–2.715)	0.025	1.998 (0.865–4.611)	0.105	1.528 (1.092–2.156)	<0.001	1.270 (0.357–4.516)	0.711
BMI > 35 kg/m^2^	1.401 (0.956–2.055)	0.084	1.509 (0.891–2.556)	0.126	1.894 (0.970–3.683)	0.06	1.219 (0.415–3.575)	0.719
ASA 3/4/5	1.385 (0.931–2.061)	0.108	1.412 (0.887–2.246)	0.146	1.166 (0.967–1.407)	0.108	1.045 (0.451–2.392)	0.929
Current smoker	0.765 (0.441–1.326)	0.340	0.505 (0.184–1.388)	0.185	0.770 (0.163–3.622)	0.740	0.960 (0.447–2.062)	0.916
Diabetes Mellitus	1.288 (0.858–1.953)	0.222	1.797 (1.060–3.045)	0.029	0.876 (0.425–1.808)	0.721	0.840 (0.226–3.131)	0.795
Hypertension	1.015 (0.718–1.434)	0.935	0.986 (0.624–1.557)	0.951	1.242 (0.671–2.298)	0.491	0.681 (0.237–1.955)	0.475
COPD	1.029 (0.416–2.543)	0.951	1.243 (0.302–5.113)	0.763	0.700 (0.213–2.294)	0.556	NE	NE
CHF	1.528 (0.661–3.535)	0.322	0.804 (0.110–5.879)	0.830	1.979 (0.752–5.208)	0.167	NE	NE
Functional status, partly dependent/ totally dependent	1.778 (0.542–5.837)	0.343	3.167 (0.843–6.925)	0.071	4.173 (0.824–7.126)	0.084	0.850 (0.447–1.616)	0.621
Operation time > 180 min	2.205 (1.452–3.356)	<0.001	2.030 (1.183–3.483)	0.01	3.380 (1.019–11.214)	0.047	0.418 (0.143–1.222)	0.111
Open Surgery	1.427 (0.841–2.420)	0.188	1.220 (0.167–8.935)	0.845	3.563 (0.884–14.369)	0.074	1.069 (0.549–2.082)	0.845
Minimally invasive surgery	1		1		1		1	
Length of hospital stay > 4 days	2.632 (1.782–3.958)	<0.001	2.966 (1.238–7.107)	0.015	5.371 (1.902–15.204)	0.004	0.962 (0.413–2.239)	0.928
Mechanical and pharmacological prophylaxis	0.616 (0.347–1.094)	0.098	0.458 (0.056–3.770)	0.468	0.235 (0.022–2.540)	0.235	0.209 (0.017–2.612)	0.225
Mechanical prophylaxis	0.593 (0.293–1.197)	0.145	0.355 (0.036–3.534)	0.377	0.205 (0.026–1.648)	0.136	0.469 (0.113–1.954)	0.299
Pharmacological prophylaxis	0.346 (0.085–1.413)	0.139	1.016 (0.059–1.7457)	0.991	0.048 (0.003–0.835)	0.037	0.251 (0.027–2.351)	0.226
No prophylaxis	1		1		1		1	

DVT = Deep vein thrombosis; BMI= Body Mass Index; ASA = American Society of Anesthesiologists Classification System; COPD = Chronic Obstructive Pulmonary Disease; CHF = Congestive Heart Failure.

## Data Availability

Data is available to all participating institutions of ACS NSQIP.
